# Impingement syndrome of the shoulder following double row suture anchor technique for arthroscopic rotator cuff repair: a case report

**DOI:** 10.4076/1752-1947-3-8109

**Published:** 2009-06-12

**Authors:** Rohit Rambani, Roger G Hackney

**Affiliations:** 1 Trauma and Orthopaedics, Leeds Teaching Hospital NHS Trust, Leeds, UK; 2 Chapel Allerton Orthopaedic Hospital, Leeds, UK

## Abstract

**Introduction:**

Arthroscopic repair of the rotator cuff is a demanding surgery. Accurate placement of anchors is key to success.

**Case presentation:**

A 38-year-old woman received arthroscopic repair of her rotator cuff using a double row suture anchor technique. Postoperatively, she developed impingement syndrome which resulted from vertical displacement of a suture anchor once the shoulder was mobilised. The anchor was removed eight weeks following initial surgery and the patient had an uneventful recovery.

**Conclusion:**

Impingement syndrome following arthroscopic repair of the rotator cuffs using double row suture anchor has not been widely reported. This is the first such case where anchoring has resulted in impingement syndrome.

## Introduction

Arthroscopic repair of a rotator cuff tear is a demanding technique. Arthroscopic repairs of rotator cuff tears have become more popular than open or mini-open repairs [1,2]. The use of double row suture anchor technique has become the standard technique among many arthroscopic shoulder surgeons [3]. Recent reports have suggested high percentages of good to excellent results even for large or massive tears with 1-3 years follow up. But the literature is still not clear about the long-term results comparing open repairs with arthroscopic repairs [2,4]. The influence of the repair technique on the failure rates and functional outcomes after open or arthroscopic rotator cuff repair remains controversial [5].

Acute impingement syndrome following rotator cuff repair has been reported to occur due to heterotopic ossification [6]. There have been no reports of acute impingement syndrome resulting from arthroscopic repair of rotator cuff from a suture anchor.

## Case presentation

**Figure 1:**
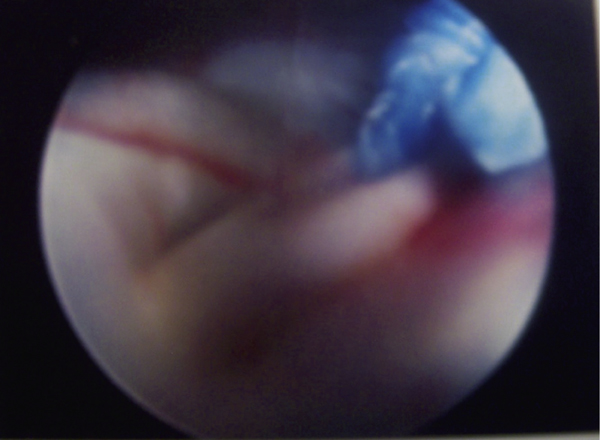
**Intra-operative photograph showing the button placed correctly**.

**Figure 2:**
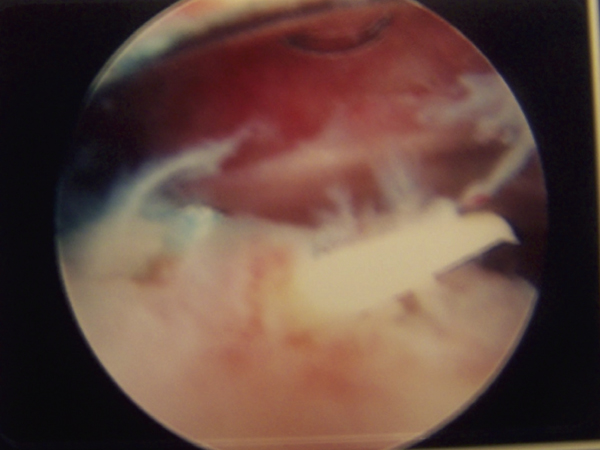
**Intra-operative photograph showing the button placed vertically ribbing the undersurface of acromion**.

A 38-year-old woman presented with a traumatic tear of her right supraspinatus measuring 1.2 cm in length and 8 mm in transverse diameter. It was repaired arthroscopically using a double row suture anchor technique. A metallic FASTIN suture anchor with orthocord (Depuy Inc. USA) was used initially followed by the Quick-T anchor fixation system (Smith & Nephew Inc Switzerland). The initial post-operative procedure was uneventful. The patient's shoulder was mobilized at six weeks. The patient started complaining of pain on abduction. This was initially treated with analgesics but when the patient did not settle the shoulder was examined using ultrasound. This showed inflammatory changes in the subacromial space and was inconclusive. The intra-operative photographs of the repair did not show any abnormality or evidence of impingement syndrome (Figure 1). The patient was taken back to theatre for diagnostic arthroscopy of the shoulder which showed the button of the Quick-T anchor lying vertically in the subacromial space rubbing the undersurface of the acromion ([Figure 2]). This was removed using a shaver without compromising the repair. Subacromial decompression was not required as there was no other evidence of impingement. Postoperatively the pain completely resolved. At follow-up three months after the second arthroscopy, the patient had full-range shoulder movement and pain was no longer present.

## Discussion

The main causes of postoperative shoulder pain include frozen shoulder [7], failure of repair [8], reflex sympathetic dystrophy [9] and subcoracoid impingement syndrome [10]. Many papers have discussed the readmission and reoperation rate after rotator cuff repairs [8] but the incidence of subacromial impingement is not documented in patients with no impingement preoperatively or intra-operatively.

The shoulder's subacromial space is of significant clinical interest due to its association with rotator cuff disease. Recent trials have suggested that subacromial decompression did not seem to significantly affect the outcome of arthroscopic rotator cuff repair on shorter follow-ups [4]. Post-operative diagnosis is usually inconclusive using ultrasonography when postoperative impingement is suspected because of inflammatory changes in the subacromial space because of surgery.

The introduction of rotator cuff MITEK anchors brought forth a fairly exclusive procedure for refixation of rotator cuff ruptures [11]. The reinsertion of the rotator cuff to their bony footprints has been suggested to have stronger and quicker healing [11,12]. Bay et al. reported an in vivo increase in subacromial space after rotator cuff repair [13].

## Conclusion

Impingement syndrome due to Quick-T suture anchors has not been reported in the literature. We report an interesting and unusual case of impingement syndrome following double row suture anchor technique.

## Competing interests

The author(s) declare that they have no competing interests.

## Consent

Written informed consent was obtained from the patient for publication of this case report and accompanying images. A copy of the written consent is available for review by the Editor-in-Chief of this journal.
